# The MT-FEM Model for Predicting Young’s Modulus of Graphene Composites with Complex Morphologies

**DOI:** 10.3390/ma18225225

**Published:** 2025-11-18

**Authors:** Xiaoxia Zhai, Huzaifa Bin Hashim, Jun Huang, Eugene Zhen Xiang Soo, Lina Luo

**Affiliations:** 1 Department of Civil Engineering, Faculty of Engineering, University of Malaya, Kuala Lumpur 50603, Malaysia; 9814005@glut.edu.cn; 2 College of Architecture and Civil Engineering, Nanning University, Nanning 530200, China; huangjun@unn.edu.cn; 3 Civil Engineering Programme, Faculty of Engineering, University Malaysia Sabah, Kota Kinabalu 88400, Malaysia; esoo@ums.edu.my; 4 Nanning Campus, Guilin University of Technology, Chongzuo 532100, China; 9814031@glutnn.cn

**Keywords:** inclusion, analytical solution, Mori-Tanaka finite element method, graphene composite, Young’s modulus

## Abstract

To address the inaccuracy of the traditional Mori-Tanaka method in predicting the Young’s modulus of graphene composites—caused by the mismatch between the assumed reinforcement shape (e.g., ellipsoidal) and the actual morphology of graphene (e.g., rectangular, oblate, wrinkled)—this paper proposes a coupled Mori–Tanaka and finite element method (MT-FEM). In this approach, the actual shape of graphene reinforcements (such as rectangular, oblate, or wrinkled forms) and their corresponding strain concentration factors are accurately obtained via finite element modeling, and then incorporated into the Mori–Tanaka framework to determine the composite Young’s modulus. The compatibility between the MT-FEM and the conventional Mori–Tanaka method is first verified through numerical examples. Furthermore, predictions by the MT-FEM show excellent agreement with existing literature results for graphene composites, with a deviation of less than 5%, confirming the reliability of the proposed method. Finally, the MT-FEM is applied to systematically investigate the influence of three graphene morphologies—rectangular, oblate, and wrinkled—on the composite Young’s modulus. Results indicate that oblate graphene yields the highest enhancement in Young’s modulus. At a graphene volume fraction of 0.03, the Young’s modulus of composites reinforced with oblate graphene reaches 4.195 GPa, which is 13.75% and 13.93% higher than those reinforced with wrinkled (3.688 GPa) and rectangular (3.682 GPa) graphene, respectively. This study provides quantitative insights and theoretical guidance for the structural design and performance optimization of graphene-reinforced composites.

## 1. Introduction

As the thinnest known material, with a monolayer thickness of merely 0.34 nm, graphene exhibits remarkable mechanical properties—including an Young’s modulus of up to 1 TPa and a tensile strength of 130 GPa—coupled with an exceptionally large specific surface area (2630 m^2^/g), as well as outstanding thermal and chemical stability [1,2,3,4]. These superior characteristics have drawn significant research attention to its application in composite materials [5]. Therefore, accurate prediction of the Young’s modulus and key performance parameters of graphene composite materials is of great significance for the structural design optimization and performance regulation of such materials [6,7,8].

Currently, the primary theoretical methods for predicting the Young’s modulus of graphene composites include the Rule of Mixtures (ROM) [9,10,11,12,13] and the Halpin–Tsai [14,15,16,17,18] model. The ROM is mainly applicable to sandwich-type representative volume element (RVE) models, while the Halpin–Tsai model is primarily used for embedded RVE configurations. However, the morphological diversity of graphene reinforcements in practical composites makes accurate characterization using a single type of RVE challenging, significantly limiting the applicability of these analytical approaches. In contrast, the Mori–Tanaka method, which does not rely on a specific RVE formulation, offers distinct advantages in predicting the elastic properties of graphene composites and has been widely adopted [19,20,21,22,23,24,25,26,27,28,29,30,31,32,33,34,35,36]. For instance, Odegard et al. [37] established a continuum-based micromechanical model for nanocomposite elasticity and validated its predictions using the Mori–Tanaka method without incorporating molecular-level details; Gary [38] systematically investigated the influence of carbon nanotube content and alignment on the composite Young’s modulus with this approach; and Dongkai Zhang et al. [39] compared the performance of finite element micromechanics and the Mori–Tanaka method in calculating the mechanical properties of carbon nanotube composites.

The theoretical foundation of the Mori–Tanaka method is based on Eshelby’s inclusion theory, which permits analytical solutions only when the inclusions are ellipsoidal in shape [40,41], or take forms derived from the ellipsoid—such as spheres, coins, and continuous fibers [42]. Although mathematically rigorous, the method has conventionally relied on the idealized assumption of ellipsoidal inclusions [43,44]. This assumption significantly deviates from the actual complex morphologies of graphene (e.g., rectangular, oblate, wrinkled), thereby limiting its predictive accuracy in practical engineering applications. For example, Cho et al. [45], in applying the Mori–Tanaka method to graphite/epoxy nanocomposites, treated the reinforcements as ellipsoidal inclusions. While they demonstrated a strong correlation between the composite modulus and the aspect ratio of the particles, the actual non-ellipsoidal morphology of graphene was not accounted for. Similarly, Jianbin Zhu et al. [46], based on Eshelby’s equivalent inclusion theory and the Mori–Tanaka method, systematically analyzed the effects of filler concentration, size, and interphase characteristics on the effective mechanical properties of composites—under the premise that graphene was modeled as an oblate spheroid.

In summary, when applying the Mori–Tanaka (M–T) method to predict the Young’s modulus of graphene composites, existing studies generally rely on oversimplified assumptions regarding reinforcement shape. The lack of dedicated analysis for realistic complex morphologies—such as rectangular, oblate, and wrinkled forms—limits prediction accuracy and hinders its applicability in engineering practice. To address this issue, this paper proposes a coupled Mori–Tanaka and finite element method (MT-FEM), developed by incorporating the structural features of graphene sheets under the condition of matrix stability at room temperature.

Building upon the conventional M–T framework, the proposed method first employs finite element modeling to accurately obtain the strain concentration factors of graphene reinforcements with actual morphologies (e.g., rectangular, oblate, wrinkled). These factors are then incorporated into the M–T theory to determine the composite Young’s modulus. The compatibility of the MT-FEM with the classical M–T method, as well as its reliability, are systematically verified through numerical examples. Furthermore, the influence of different graphene shapes on the composite modulus is thoroughly investigated.

This approach not only overcomes the idealized shape restrictions inherent in the traditional M–T method, enabling accurate characterization of the mechanical contribution of reinforcements with arbitrary morphology, but also avoids complex modeling steps—such as random inclusion placement and spatial arrangement optimization—required in pure finite element simulations. Thus, the proposed method offers a more reliable theoretical reference and quantitative basis for the structural design and performance optimization of graphene-reinforced composites.

## 2. The Fundamental Theory

### 2.1. Mori-Tanaka Method

**Figure 1:**
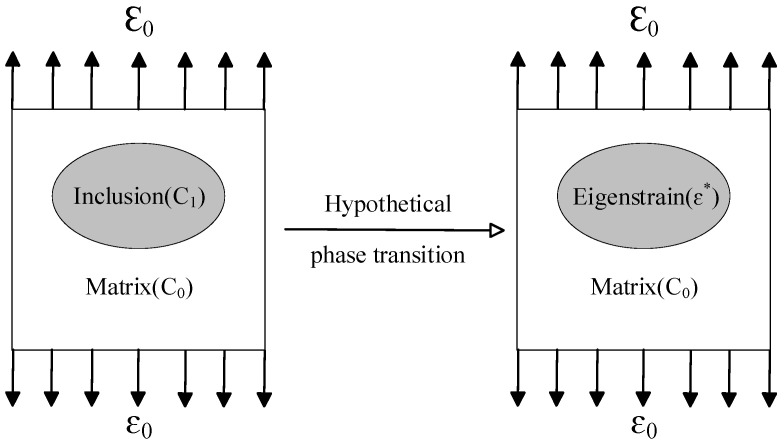
Eshelby’s equivalent inclusion principle. Here, the eigenstrain is denoted by the symbol $\varepsilon^{*}$.

Eshelby’s equivalent inclusion principle is illustrated in Figure 1. When an ellipsoidal inclusion with a modulus of $C_{1}$ is embedded in an infinite homogeneous medium with a modulus of $C_{0}$, and the medium is subjected to a far-field uniform macroscopic external load of $\varepsilon_{0}$, the inclusion is hypothetically transformed into a material identical to the matrix through a phase transformation. The eigenstrain $\varepsilon^{*}$ required for this phase transformation is then introduced. Furthermore, by applying the superposition principle and the constitutive relations of the inclusion and the matrix, the relationship between the average strain inside the inclusion and the far-field uniform external load is derived. This enables the acquisition of an analytical solution that facilitates the calculation of the single-inclusion problem.

.

**Figure 2:**
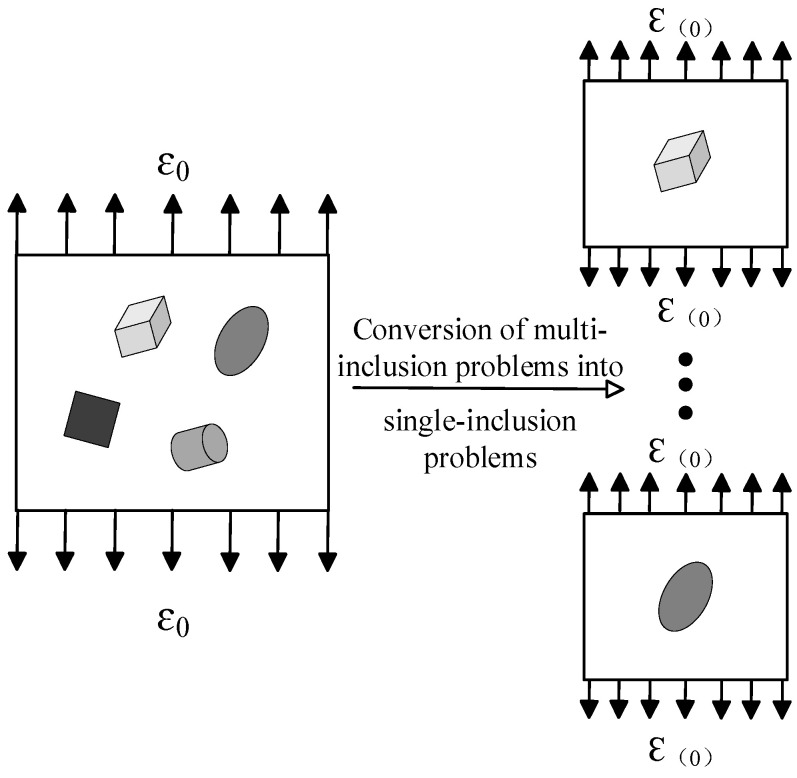
Single-Inclusion Simplification for Multi-Inclusion Problems via Mori-Tanaka Method. Here, $\varepsilon_{0}$ denotes the applied far-field strain in the multi-inclusion scenario, while $\varepsilon_{\left(0\right)}$ denotes the equivalent applied far-field strain for a single inclusion. They differ due to the conversion of boundary conditions.

The Mori-Tanaka method posits that for the representative element of a composite material, the specific strain (denoted as $\varepsilon_{\left(0\right)}$) acting around a particular inclusion differs from the macroscopic strain (denoted as $\varepsilon_{0}$) applied at a remote location—this difference arises due to the presence of other inclusions. Based on this observation, when the method converts a multi-inclusion problem into a single-inclusion problem, it defines the far-field macroscopic constant strain in the single-inclusion problem as the average strain of the composite’s matrix. Notably, the average strain of the composite’s matrix is an unknown quantity that needs to be determined. The method further assumes that the average strain of the composite’s matrix is the volume-averaged value of the average strains of each constituent material. Under this assumption, the relationship between the macroscopic constant strain of the multi-inclusion matrix and the matrix average strain of the composite containing only this single inclusion is illustrated in Figure 2.

There are many forms of the Mori-Tanaka (M-T) expression, but two are commonly used [39]. One of them was introduced by Benveniste [47] in 1987, and the formula is as follows:

(1) $$\bar{C}=C_{0}+\sum_{n=1}^{n}v_{frn}\left(C_{n}-C_{0}\right):T_{n}:{\left(v_{fr0}J+\sum_{n=1}^{n}v_{frn}T_{n}\right)}^{-1}$$

Here, it is assumed that there are n types of inclusions, where n = 1, 2, 3 …; in the formula, $\bar{C}$ denotes the effective stiffness of the composite material, $C_{n}$ represents the stiffness tensor of the n-th phase inclusion, and $v_{frn}=\frac{v_{n}}{v}$ stands for the volume fraction of the n-th phase material—with $v_{n}$ being the volume of the n-th phase material and v being the total volume of the composite material; when n=0, $v_{fr0}$ represents the volume fraction of the matrix material.

where

(2) $$T_{n}={\left[J+E_{n}:C_{0}^{-1}:\left(C_{n}-C_{0}\right)\right]}^{-1}$$

In the formula, J is a fourth-order unit tensor, and $E_{n}$ is the Eshelby tensor of the n-th phase inclusion, whose values can be found in reference [42].

The other is a simpler and more universal expression form derived with the help of the strain concentration factor $A_{n}$, and the expression is as follows [47,48,49]:

(3) $$\bar{C}=C_{0}+\sum_{n=1}^{n}v_{frn}\left(C_{n}-C_{0}\right):A_{n}$$

Among them, the strain concentration factor $A_{n}$ can be obtained using Eshelby’s solution to the single-inclusion problem under different approximation conditions, and different approximation methods have thus been derived.

### 2.2. Common Forms of the Eshelby Tensor

**Figure 3:**
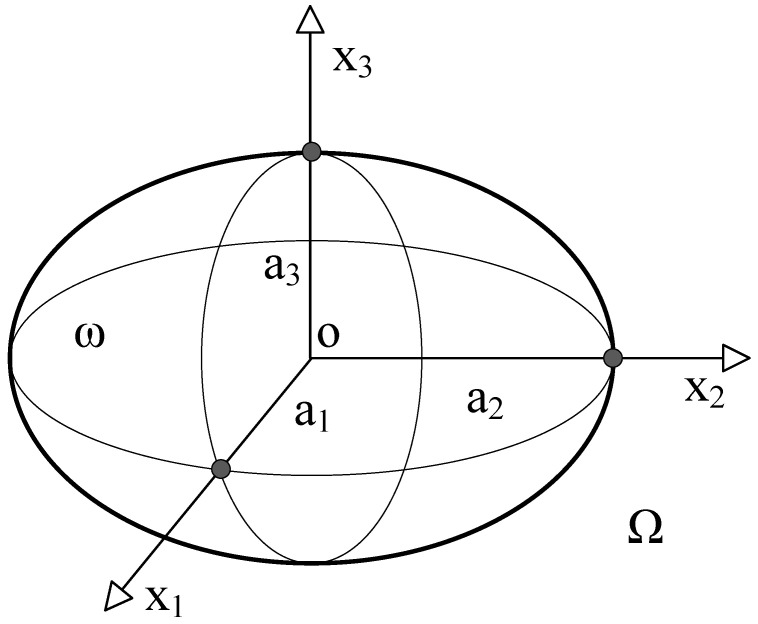
Schematic Diagram of Inclusion ω in an Infinite Space Ω.

Within the infinite domain Ω (assumed to be an isotropic matrix), there is an ellipsoidal inclusion domain ω; the radii of the three principal axes of the ellipsoidal inclusion are, respectively, as shown in Figure 3. Therefore, all points $x_{1},x_{2},x_{3}$ inside the ellipsoidal inclusion must satisfy the following relationship:

(4) $$\frac{x_{1}^{2}}{a_{1}^{2}}+\frac{x_{2}^{2}}{a_{2}^{2}}+\frac{x_{3}^{2}}{a_{3}^{2}}\leq 1$$

For an ellipsoidal inclusion, if the strain applied at a remote location is uniform, it can be proven that the strain inside the ellipsoidal inclusion is also uniform. The relationship between the strain $\varepsilon_{\left(\omega \right)}$ inside the inclusion and the uniform strain $\varepsilon_{0}$ applied at the remote location can be expressed as follows.

(5) $$\varepsilon_{(\omega \omega )i}=E_{inkl}\varepsilon_{0kl}$$

Among them, $E_{inkl}$ is referred to as the Eshelby tensor. For the subscript i,n, it is symmetric with the subscript k,l, i.e., $E_{inkl}=E_{nikl}=E_{inlk}$; however, it is generally not symmetric with respect to the subscript $\left(i,n\right)$ and subscript $\left(k,l\right)$, i.e., $E_{inkl}\neq E_{klin}$. In linear elasticity problems, the Voigt notation is utilized, whereby the fourth-order elasticity tensor is represented in a compact 6 × 6 matrix form. Therefore, the fourth-order tensor $E_{inkl}$ is converted into a matrix $E_{IJ}$ by mapping each pair of indices (i, k) and (n, l) to a single index according to the Voigt rule.

For isotropic materials, the stiffness matrix $E_{IJ}$ simplifies to:

(6) $$E_{IJ}=\left[\begin{array}{lllllll} E_{11} & E_{12} & E_{13} & 0 & 0 & 0\\ E_{21} & E_{22} & E_{23} & 0 & 0 & 0 & \\ E_{31} & E_{32} & E_{33} & 0 & 0 & 0\\ 0 & 0 & 0 & 2E_{44} & 0 & 0\\ 0 & 0 & 0 & 0 & 2E_{55} & 0\\ 0 & 0 & 0 & 0 & 0 & 2E_{66} \end{array}\right]$$

Shen Guanlin et al. [42] derived the Eshelby tensors for four commonly used inclusion shapes based on ellipsoidal inclusions. These shapes are, respectively: prolate ellipsoidal inclusions (where $a_{2}=a_{3}=a$ and $x_{1}$ are referred to as the rotation axes, with an aspect ratio of $\rho =\frac{a_{1}}{a}$), spherical inclusions ($a_{1}=a_{2}=a_{3}$), long fiber-shaped inclusions ($a_{2}=a_{3}=a$, $a_{1}\to \infty$), and coin-shaped inclusions ($a_{2}=a_{3}=a$, $a_{1}\to 0$). For typical monolayer graphene sheets, their microscopic morphology more closely resembles a rectangular cuboid. In the cases of long fibers and coin-shaped inclusions, an extreme value is assumed for one of the morphological parameters, which can lead to the adoption of such extreme values when modeling graphene inclusions that are actually closer to a cuboid shape. Here, we select both ellipsoidal and spherical inclusions for comparative analysis.

#### 2.2.1. Ellipsoidal (Where $a_{2}=a_{3}=a$ and $x_{1}$ Are Referred to as the Rotation Axes, with an Aspect Ratio of $\rho =\frac{a_{1}}{a}$)

When the ellipsoidal inclusion model is adopted, the non-zero parameters are listed below [42].

(7) $$\begin{array}{l} E_{11}=\frac{1}{2(1-\mu_{0})}\left[4-2\mu_{0}-\frac{2}{\left(1-\rho^{2}\right)}\right]+\frac{1}{2(1-\mu_{0})}\left[2\mu_{0}-4+\frac{3}{\left(1-\rho^{2}\right)}\right]g\\ E_{22}=E_{33}=-\frac{3}{8(1-\mu_{0})}\frac{\rho^{2}}{\left(1-\rho^{2}\right)}+\frac{1}{4(1-\mu_{0})}\left[1-2\mu_{0}-\frac{9}{4(1-\rho^{2})}\right]g\\ E_{23}=E_{32}=\frac{1}{8(1-\mu_{0})}\left[1-\frac{1}{\left(1-\rho^{2}\right)}\right]+\frac{1}{16(1-\mu_{0})}\left[-4(1-2\mu_{0})+\frac{3}{\left(1-\rho^{2}\right)}\right]g\\ E_{21}=E_{31}=\frac{1}{2(1-\mu_{0})}\frac{\rho^{2}}{\left(1-\rho^{2}\right)}-\frac{1}{4(1-\mu_{0})}\left[(1-2\mu_{0})+\frac{3\rho^{2}}{\left(1-\rho^{2}\right)}\right]g\\ E_{12}=E_{13}=\frac{1}{2(1-\mu_{0})}\left[-(1-2\mu_{0})+\frac{1}{\left(1-\rho^{2}\right)}\right]+\frac{1}{4(1-\mu_{0})}\left[2(1-2\mu_{0})-\frac{3}{\left(1-\rho^{2}\right)}\right]g\\ E_{44}=\frac{1}{8(1-\mu_{0})}\frac{\rho^{2}}{\left(1-\rho^{2}\right)}+\frac{1}{16(1-\mu_{0})}\left[4(1-2\mu_{0})+\frac{3}{\left(1-\rho^{2}\right)}\right]g\\ E_{55}=E_{66}=\frac{1}{4(1-\mu_{0})}\left[(1-2\mu_{0})+\frac{1+\rho^{2}}{\left(1-\rho^{2}\right)}\right]-\frac{1}{8(1-\mu_{0})}\left[(1-2\mu_{0})+\frac{3(1+\rho^{2})}{\left(1-\rho^{2}\right)}\right]g \end{array}$$

Among them, $\mu_{0}$ denotes the Poisson’s ratio of the matrix, and the values of ρ and g are given as follows:

(8) $$\begin{array}{l} when\;\rho >1:\\ g=\frac{\rho }{{\left(\rho^{2}-1\right)}^{3/2}}\left[\rho {\left(\rho^{2}-1\right)}^{1/2}-\arccos h\left(\rho \right)\right];\\ when\;\rho <1:\\ g=\frac{\rho }{{\left(1-\rho^{2}\right)}^{3/2}}\left[\arccos\left(\rho \right)-\rho {\left(1-\rho^{2}\right)}^{1/2}\right]. \end{array}$$

#### 2.2.2. Spherica ($a_{1}=a_{2}=a_{3}$)

When the spherica inclusion model is adopted, the non-zero parameters are listed below [42].

(9) $$\begin{array}{l} E_{11}=E_{22}=E_{33}=\frac{7-5\mu_{0}}{15(1-\mu_{0})}\\ E_{12}=E_{13}=E_{21}=E_{31}=E_{23}=E_{32}=\frac{5\mu_{0}-1}{15(1-\mu_{0})}\\ E_{44}=E_{55}=E_{66}=\frac{4-5\mu_{0}}{15(1-\mu_{0})} \end{array}$$

### 2.3. Numerical Method for Calculating Inclusion Strain Factors

#### 2.3.1. Principle of Numerical Calculation

Based on the derivation and solution for ellipsoidal inclusions, there are currently relatively mature and easy-to-calculate explicit solutions for ellipsoidal, spherical, coin-shaped, and long fiber-shaped inclusions. However, for single-layer graphene—an angular flaky inclusion that belongs to the cuboidal inclusion category when its planar size is small—there is no convenient explicit solution available for calculation yet. Nevertheless, the finite element method can be extended to composite materials with different inclusion shapes.

The M-T finite element method uses a matrix material of infinite size (5–10 times the size of the inclusion) to enclose a typical graphene reinforcement. Uniform boundary conditions are applied to establish a boundary value problem, which is then solved using the finite element method to obtain the strain concentration factor A; finally, the effective modulus is derived.

As can be seen from Equation (5), the strain concentration factor A describes the linear relationship between the average strain inside the inclusion and the far-field macroscopic constant strain in the composite. Each element $A_{ij}$ in the matrix represents the influence coefficient of the i-th strain component generated inside the inclusion when the j-th strain component is applied in the far field. Assuming that graphene and the matrix are isotropic materials, the final form of matrix $A_{ij}$ is as follows:

(10) $$A_{ij}=\left[\begin{array}{llllll} A_{11} & A_{12} & A_{13} & 0 & 0 & 0\\ A_{21} & A_{22} & A_{23} & 0 & 0 & 0\\ A_{31} & A_{32} & A_{33} & 0 & 0 & 0\\ 0 & 0 & 0 & A_{44} & 0 & 0\\ 0 & 0 & 0 & 0 & A_{55} & 0\\ 0 & 0 & 0 & 0 & 0 & A_{66} \end{array}\right]$$

Each column of the strain concentration factor matrix corresponds to an independent far-field strain condition, and the detailed mapping rules are shown in Table 1 below.

#### 2.3.2. Establishment of the Finite Element Model

**Figure 4:**
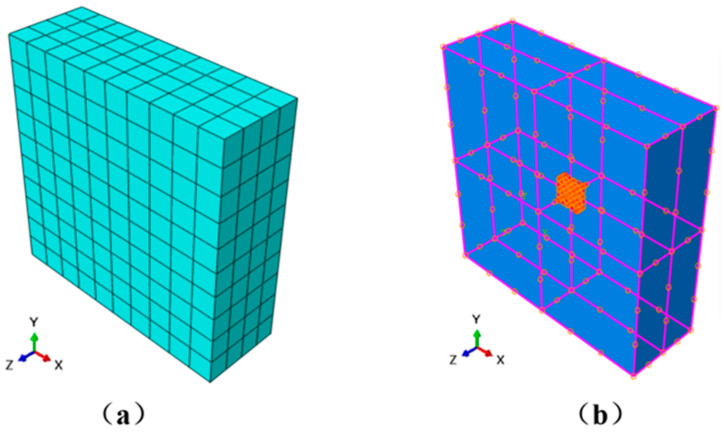
Finite element mesh of the model: (**a**) the meshing of the matrix and cell selection (**b**) the bonding of the two phases.

The finite element modeling and analysis in this study were conducted using ABAQUS 6.14. In the model, the matrix was simulated using the built-in 8-node linear brick element (C3D8), while the graphene reinforcement was represented by either the 8-node brick element (C3D8) or the 4-node shell element (S4), depending on the specific modeling requirements. An ideally smooth interface was assumed between the graphene and the polymer matrix, with interfacial interactions neglected. Through the built-in “embedded region” constraint, the matrix was defined as the host and the graphene reinforcement as the embedded guest, thereby establishing an analytical model for determining the strain factors of a single graphene inclusion. The corresponding finite element mesh and connectivity are illustrated in Figure 4.

#### 2.3.3. Application of Boundary Conditions

For the applied far-field strain, the $\varepsilon^{\infty }=\left[\varepsilon_{11}^{\infty },\varepsilon_{22}^{\infty },\varepsilon_{33}^{\infty },\varepsilon_{12}^{\infty },\varepsilon_{13}^{\infty },\varepsilon_{23}^{\infty }\right]$, the average strain in the inclusion $\varepsilon^{avg}=\left[\varepsilon_{11}^{avg},\varepsilon_{22}^{avg},\varepsilon_{33}^{avg},\varepsilon_{12}^{avg},\varepsilon_{13}^{avg},\varepsilon_{23}^{avg}\right]$ was obtained. The components of the strain factor matrix $A_{i1}$ were calculated using the following formulas.

(11) $$A_{11}=\frac{\varepsilon_{11}^{avg}}{\varepsilon_{11}^{\infty }},A_{21}=\frac{\varepsilon_{22}^{avg}}{\varepsilon_{11}^{\infty }},A_{31}=\frac{\varepsilon_{33}^{avg}}{\varepsilon_{11}^{\infty }},A_{41}=\frac{2\varepsilon_{12}^{avg}}{\varepsilon_{11}^{\infty }},A_{51}=\frac{2\varepsilon_{13}^{avg}}{\varepsilon_{11}^{\infty }},A_{61}=\frac{2\varepsilon_{23}^{avg}}{\varepsilon_{11}^{\infty }}$$

**Figure 5:**
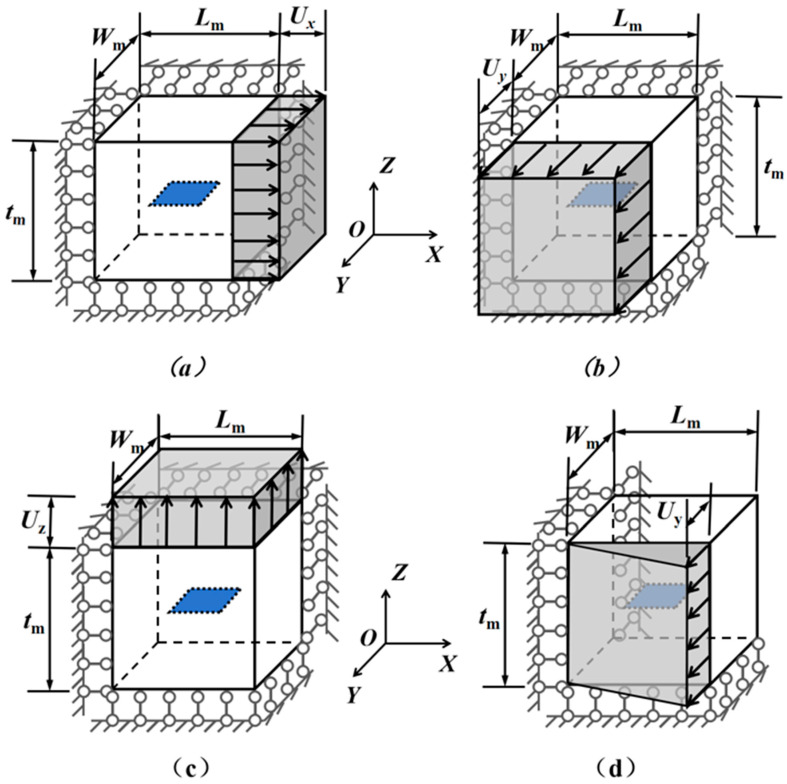

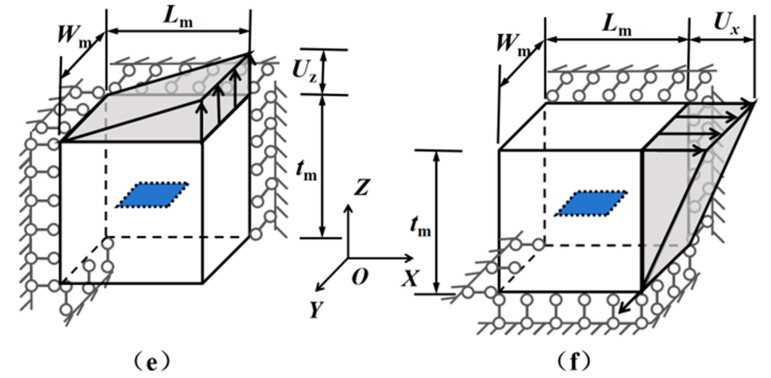
Boundary Conditions (**a**) $A_{i1}$; (**b**) $A_{i2}$; (**c**) $A_{i3}$; (**d**) $A_{i4}$; (**e**) $A_{i5}$; (**f**) $A_{i6}$.

The boundary conditions used to determine each column of the strain factor matrix $A_{ij}$ for the graphene inclusion are shown in Figure 5.

The dimensions and directional displacements of the graphene and matrix in the model are defined as follows: the graphene dimensions include length ($L_{g}$), width ($W_{g}$), and thickness ($t_{g}$); the matrix dimensions include length ($L_{m}$), width ($W_{m}$), and thickness ($t_{m}$). The axial displacements along the X, Y, and Z axes are denoted as $U_{x}$, $U_{y}$, and $U_{z}$, respectively. All related symbols in this paper are consistent with the above definitions, and all involved dimensions are expressed in nanometers (nm). These notations remain consistent throughout the text. Below we describe them in detail:

(a) The boundary condition corresponding to $A_{i1}$ is illustrated in Figure 5a. Displacement in the X-direction is constrained on the plane X=0, while a small displacement $U_{x}$ is applied on the plane $X=L_{m}$. In addition, displacements in the Y- and Z-directions are restricted on the planes Y=0 and Z=0, respectively. All other surfaces remain unconstrained.
(b) The boundary condition corresponding to $A_{i2}$ is illustrated in Figure 5b. Displacement in the Y-direction is constrained on the plane Y=0, while a small displacement $U_{y}$ is applied on the plane $Y=W_{m}$. In addition, displacements in the X- and Z-directions are restricted on the planes X=0 and Z=0, respectively. All other surfaces remain unconstrained.
(c) The boundary condition corresponding to $A_{i3}$ is illustrated in Figure 5c. Displacement in the Z-direction is constrained on the plane Z=0, while a small displacement $U_{z}$ is applied on the plane $Z=t_{m}$. In addition, displacements in the X- and Y-directions are restricted on the planes X=0 and Y=0, respectively. All other surfaces remain unconstrained.
(d) The boundary condition corresponding to $A_{i4}$ is illustrated in Figure 5d. Specifically, the X-direction displacement was fixed on the plane X=0, and the Z-direction displacement was fixed on the plane Z=0. The Y-direction displacement was constrained along the model’s *Z*-axis, defined by the intersection line of planes X=0 and Y=0. A prescribed Y-direction displacement, $U_{y}$, was applied at the intersection of the surfaces $X=L_{m}$ and $Y=W_{m}$. All other boundaries remained free of constraints.
(e) The boundary condition corresponding to $A_{i5}$ is illustrated in Figure 5e. Specifically, the X-direction displacement was fixed on the plane X=0, and the Y-direction displacement was fixed on the plane Y=0. The Z-direction displacement was constrained along the model’s *Y*-axis, defined by the intersection line of planes X=0 and Z=0. A prescribed Z-direction displacement, $U_{z}$, was applied at the intersection of the surfaces $X=L_{m}$ and $Z=t_{m}$. All other boundaries remained free of constraints.
(f) The boundary condition corresponding to $A_{i6}$ is illustrated in Figure 5f. Specifically, the Y-direction displacement was fixed on the plane Y=0, and the Z-direction displacement was fixed on the plane Z=0. The X-direction displacement was constrained along the model’s *Y*-axis, defined by the intersection line of planes X=0 and Z=0. A prescribed Z-direction displacement, $U_{x}$, was applied at the intersection of the surfaces $X=L_{m}$ and $Z=t_{m}$. All other boundaries remained free of constraints.

The displacements in the three coordinate directions must satisfy the following equations:

(12) $$U_{x}=\varepsilon_{11}^{\infty }L_{m};U_{y}=\varepsilon_{11}^{\infty }W_{m};U_{z}=\varepsilon_{11}^{\infty }t_{m};$$

## 3. Results and Discussion

### 3.1. Comparison Between the MT-FEM Method and the M-T Method

To verify the reliability of the Mori-Tanaka (M-T) finite element method, ellipsoidal inclusions and spherical inclusions with existing analytical solutions were selected as research objects in this example. For these two inclusions of different shapes, the M-T finite element method and the traditional M-T method were, respectively, used for calculation. By comparing the differences in the Young’s modulus Ecx obtained by the two methods, the calculation reliability of the M-T finite element method under different inclusion shapes was further demonstrated.

Graphene [50] has an Young’s modulus of $E_{g}=829\;\mathrm{GPa}$ and a Poisson’s ratio of $\mu_{g}=0.344$. The matrix is an isotropic epoxy-based material [51], with an Young’s modulus of $E_{m}=3\;\mathrm{GPa}$ and a Poisson’s ratio of $\mu_{m}=0.34$. When the volume fractions of the reinforcement are 0.01, 0.02, 0.03, 0.04, and 0.05, respectively, the comparison of calculation results using two commonly used inclusion shape parameters is shown in Table 2.

To ensure consistent reinforcement volume, the ellipsoidal and spherical inclusions are assumed to possess the same volume as a typical monolayer graphene sheet with dimensions *L_g_* × *W_g_* × *t_g_* = 1 × 1 × 0.34 nm. The calculated radius of the spherical inclusion is 0.433 nm, while the ellipsoid has a semi-minor axis of 0.34 nm and semi-major axes of 0.4887 nm. These dimensions are derived using the parameter selection method described in Section 2.2 for the Mori–Tanaka (M–T) method. For the M-T finite element (MT-FEM) approach, the strain concentration factors are obtained following the methodology in Section 2.3, yielding factor $A_{E}$ for the ellipsoidal inclusion and factor $A_{S}$ for the spherical inclusion. Their respective non-zero parameters are given by Equations (13) and (14) below.

(13) $$\begin{array}{l} A_{11}^{E}=0.0054;A_{22}^{E}=0.0043;A_{33}^{E}=0.0042;\\ A_{12}^{E}=A_{13}^{E}=A_{21}^{E}=A_{23}^{E}=A_{31}^{E}=A_{32}^{E}=0.0007;\\ A_{44}^{E}=0.0026;A_{55}^{E}=0.0005;A_{66}^{E}=0.0002; \end{array}$$

(14) $$\begin{array}{l} A_{11}^{S}=0.0047;A_{22}^{S}=0.00413;A_{33}^{S}=0.0074;\\ A_{12}^{S}=A_{13}^{S}=-0.001;\\ A_{21}^{S}=-0.0007;A_{23}^{S}=-0.0012;\\ A_{31}^{S}=-0.0009;A_{32}^{S}=-0.0008;\\ A_{44}^{S}=0.00412;A_{55}^{S}=0.001;A_{66}^{S}=0.0006; \end{array}$$

Based on the data in Table 2, whether for prolate ellipsoidal inclusions or spherical inclusions, the predicted values of Young’s modulus Ecx obtained by the M-T finite element method are slightly larger than those from the traditional M-T method. However, both methods exhibit a consistent variation trend: as the graphene volume fraction gradually increases from 0.01 to 0.05, the predicted Young’s modulus values increase continuously.

Regarding the deviation, for prolate ellipsoidal inclusions, the deviation gradually rises from 0.853% (at a volume fraction of 0.01) to 4.006% (at a volume fraction of 0.05); for spherical inclusions, the deviation increases from 0.891% to 4.304%. Although the deviation increases with the rise in volume fraction, all deviations are controlled within 5%.

In conclusion, the MT-FEM can accurately reflect the variation law of the Young’s modulus of graphene composites with the reinforcement volume fraction, and thus can be applied to the prediction of the effective mechanical properties of graphene composites.

### 3.2. Comparison with Results from Other Studies

To validate the reliability of the proposed Mori–Tanaka and finite element coupling method (MT-FEM), the predicted Young’s modulus of graphene composites obtained by this approach is compared with experimental data from existing literature.

Specifically, following the modeling procedures and boundary conditions described in Section 2.3, this example applies the MT-FEM to extract the strain concentration factors of the reinforcement based on the graphene dimensions provided in the literature. These factors are then substituted into Equation (3) to compute the Young’s modulus of the graphene composites. The resulting predictions are compared with experimental results from published studies, as detailed in Table 3.

As shown in the table, the MT-FEM predictions consistently align well with the experimental data across varying graphene volume fractions (Vg), dimensional parameters (Lg, Wg, tg), and modulus values (Em, Eg), with all deviations remaining within 5%. The maximum observed deviation is 4.48%, while the minimum is −0.5%. For instance, in Case 4, the experimental result is 3.3 GPa, and the MT-FEM prediction is 3.448 GPa, corresponding to a deviation of 4.48%. In Case 1, the measured value of 0.2 GPa differs from the predicted 0.201 GPa by only 0.5%. These results indicate that the MT-FEM method is capable of accurately predicting the Young’s modulus of graphene composites, regardless of variations in graphene volume fraction, geometry, or the modulus combination of the matrix and reinforcement.

Through comparison with multiple sets of experimental data from the literature, it is demonstrated that the proposed MT-FEM method achieves high predictive accuracy for the Young’s modulus of graphene composites, with deviations consistently below 5%. This fully validates the reliability of the method and confirms its utility as an effective theoretical tool for analyzing the mechanical properties of graphene composites.

### 3.3. Influence of Graphene Shape on the Young’s Modulus of Composites

In this example, graphene sheets with a uniform thickness of 0.34 nm are selected. Under the condition of constant surface area, a systematic investigation is conducted into the influence of three surface morphologies—rectangular, oblate, and wrinkled—on the composite Young’s modulus. The specific material parameters for the graphene and matrix are provided in Section 3.1.

**Figure 6:**
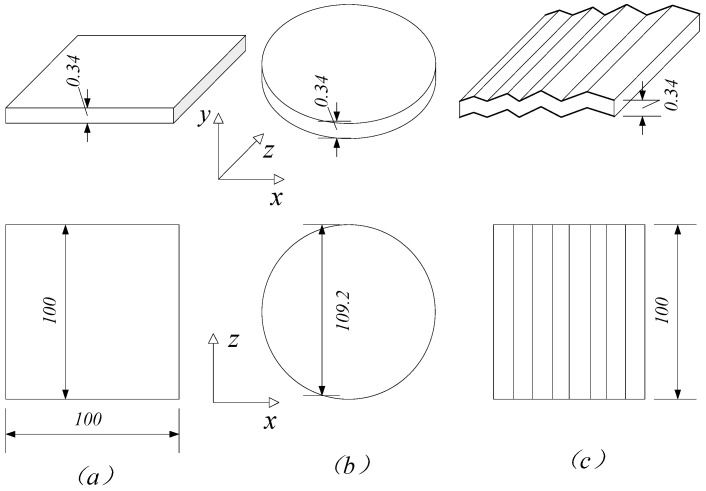
Graphene morphologies: (**a**) Rectangular; (**b**) Oblate; (**c**) Wrinkled.

The geometric dimensions of the reinforcements are defined as follows: the rectangular graphene is assigned side lengths of *L_g_* = *W_g_* = 100 nm. Based on the same surface area, the equivalent radius of the oblate graphene is calculated to be 56.4 nm. To maintain consistency in the matrix geometry, the case with a wrangle amplitude of 1° is analyzed, whose geometric model is illustrated in Figure 6.

**Figure 7:**
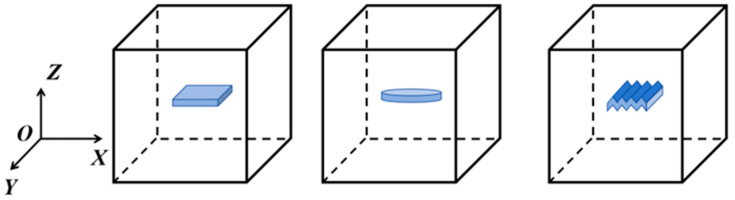
Schematic of the graphene arrangement within the matrix.

The spatial distribution patterns of these three graphene shapes within the matrix are illustrated in Figure 7, showing that the rectangular, oblate, and wrinkled graphene are all uniformly dispersed in the matrix material with aligned orientations.

The modeling data for the wrinkled graphene are presented in Table 4. This table depicts the macroscopic morphology of the wrinkled graphene, along with the numbers (“CP No.”) and coordinates of the corresponding control points which define the profile.

The computational results are presented in Table 5.

The results indicate that the Young’s modulus (Ecx) of the composites increases monotonically with the volume fraction (Vg) for all three graphene morphologies (wrinkled, circular, and rectangular). Among them, the circular graphene consistently delivers the most effective reinforcement. For instance, at Vg = 0.03, the Ecx value for the circular morphology reaches 4.195 GPa, significantly higher than those of the wrinkled (3.688 GPa) and rectangular (3.682 GPa) geometries. As Vg increases from 0.005 to 0.03, the relative enhancement advantage of circular graphene expands from approximately 2.5% to nearly 14%, while the absolute difference in modulus increases from about 0.08 GPa to 0.51 GPa. This clearly demonstrates that the circular graphene, owing to its geometric advantages, can more effectively enhance the composite stiffness, and this superiority becomes more pronounced with increasing volume fraction.

**Figure 8:**
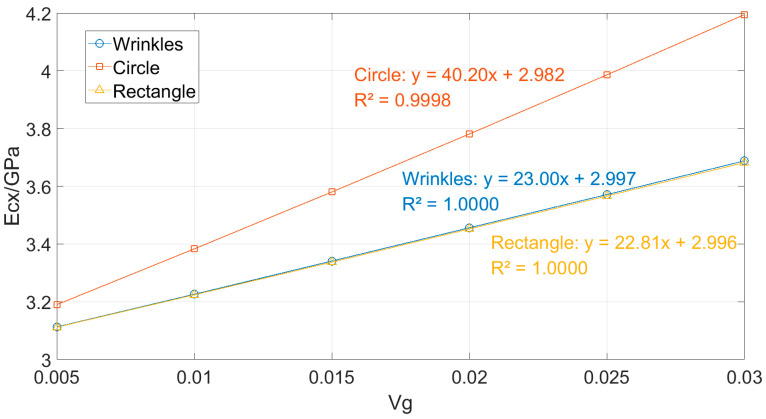
Variation in Ecx with Graphene Volume Fraction for Different Shapes and Their Linear Fitting Curves.

The data from Table 5 are fitted and plotted in Figure 8.

The linear fitting results in Figure 8 show that the R^2^ values for all three shapes are close to 1, indicating a strong linear correlation between Vg and the Young’s modulus. Among them, the circular shape exhibits the highest fitting slope (approximately 40.20), significantly greater than those of the wrinkled (23.00) and rectangular (22.81) shapes, reflecting its fastest growth rate of Young’s modulus with increasing Vg and the steepest corresponding curve. In contrast, the slopes of the wrinkled and rectangular shapes are similar, implying nearly identical growth rates. This difference in slope reflects the shape-dependent sensitivity to Vg variation: the circular shape is the most sensitive to changes in Vg, where even a slight increase in volume fraction leads to a more pronounced improvement in modulus, which is closely related to its higher stress transfer efficiency attributable to its geometry.

## 4. Conclusions

To address the limitations of the traditional Mori-Tanaka (M-T) method in accurately predicting the Young’s modulus of composites due to the mismatch between idealized reinforcement shapes (e.g., ellipsoids) and actual graphene morphologies (e.g., rectangular, oblate, wrinkled), this study proposes a coupled Mori–Tanaka and finite element method (MT-FEM). By incorporating strain concentration factors derived from finite element modeling of realistic graphene morphologies into the M-T theoretical framework, this approach enables efficient and accurate prediction of the composite Young’s modulus.

The main findings are as follows:

(a) The MT-FEM method demonstrates consistent trends in modulus prediction with the conventional M-T approach, confirming their theoretical compatibility;
(b) The deviations between MT-FEM predictions and experimental data from the literature are all within 5%, indicating high reliability;
(c) A systematic comparison of three typical graphene morphologies reveals that the oblate shape provides the most significant reinforcement effect—at a volume fraction of 0.03, the composite modulus reaches 4.195 GPa, which is 13.75% and 13.93% higher than those reinforced by wrinkled (3.688 GPa) and rectangular (3.682 GPa) graphene, respectively.

The proposed method innovatively overcomes the idealized shape limitations of the traditional M-T approach, enabling accurate characterization of the mechanical contribution of graphene with arbitrary morphology. Moreover, it avoids the modeling complexities associated with pure finite element simulations, such as random reinforcement distribution and spatial arrangement optimization. This study provides a reliable theoretical foundation and quantitative basis for the structural design and performance optimization of graphene composites.

## Figures and Tables

**Table 1:** The elements of the strain factor A column are related to the far-field strain boundary conditions and their corresponding physical meanings.

No.	Far-Field Uniform Strain $\varepsilon_{0}$	Corresponding Matrix Element	Physical Meaning
1	$\varepsilon_{11}$	$A_{i1}$	The deformation response of the inclusion under far-field uniaxial tension (in the x-direction).
2	$\varepsilon_{22}$	$A_{i2}$	The deformation response of the inclusion under far-field uniaxial tension (in the y-direction).
3	$\varepsilon_{33}$	$A_{i3}$	The deformation response of the inclusion under far-field uniaxial tension (in the z-direction).
4	$\varepsilon_{12}$	$A_{i4}$	The deformation response of the inclusion under far-field shear in the xy-plane.
5	$\varepsilon_{23}$	$A_{i5}$	The deformation response of the inclusion under far-field shear in the yz-plane.
6	$\varepsilon_{31}$	$A_{i6}$	The deformation response of the inclusion under far-field shear in the xz-plane.

**Table 2:** Comparison of Ecx Calculated by the MT-FEM and M-T Methods.

No.	Vg	Ellipsoidal Ecx [GPa]			Spherica Ecx [GPa]		
		M-T (1)	MT-FEM (2)	$\mathrm{Deviation}\;(\%)\;(\frac{\left(2\right)-\left(1\right)}{\left(1\right)})$	M-T (3)	MT-FEM (4)	$\mathrm{Deviation}\;(\%)\;(\frac{\left(4\right)-\left(3\right)}{\left(3\right)})$
1	0.01	3.023	3.049	0.853	3.005	3.032	0.891
2	0.02	3.046	3.098	1.679	3.010	3.064	1.762
3	0.03	3.070	3.147	2.447	3.015	3.096	2.616
4	0.04	3.092	3.196	3.254	3.020	3.128	3.453
5	0.05	3.115	3.245	4.006	3.024	3.160	4.304

**Table 3:** Comparison between MT-FEM Predictions and Experimental Data.

No.	Vg (%)	Graphene Size		Em [GPa]	Eg [GPa]	Test Result (1)	Present Result (2)	Deviation (%)
		Lg = Wg (nm)	tg (nm)					$\left(\frac{\left(2\right)-\left(1\right)}{\left(1\right)}\right)$
1 [52]	0.3	1000	0.8	0.1	1000	0.2	0.201	0.5
2 [52]	0.6	1000	0.8	0.1	1000	0.25	0.246	−1.6
3 [52]	1.8	1000	0.8	0.1	1000	1.04	1.029	−1.1
4 [45]	0.523	1000	200	3.27	1153	3.3	3.448	4.48
5 [45]	0.523	1000	14.3	3.27	1153	3.65	3.559	−2.49
6 [53]	0.6	15,000	7	2.72	1000	2.8	2.75	−1.78
7 [53]	1.21	15,000	7	2.72	1000	2.94	2.895	−1.53
8 [53]	1.82	15,000	7	2.72	1000	3.03	2.984	−1.52
9 [53]	2.44	15,000	7	2.72	1000	3.11	3.073	−1.19
10 [53]	3.06	15,000	7	2.72	1000	3.24	3.163	−2.38
11 [53]	3.69	15,000	7	2.72	1000	3.35	3.255	−2.84
12 [17]	2	10,000	1.7	1.0182	1000	1.4800	1.452	−1.89
13 [17]	4	10,000	1.7	1.0182	1000	1.8034	1.868	−1.58

**Table 4:** Coordinate Table of Control Points for Wrinkled Morphology.

Wrinkle Profile			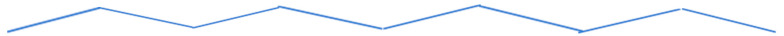							
CP No.		1	2	3	4	5	6	7	8	9
Coordinate	X	0	12.498	24.996	37.494	49.992	62.490	74.988	87.487	99.985
	Y	0	0.218	0	0.218	0	0.218	0	0.218	0

**Table 5:** Predicted Ecx of Graphene Composites with Different Shapes at Various Volume Fractions.

No.	Vg	Ecx [GPa]			Absolute Difference (GPa)		Relative Difference (%)	
		Oblate (1)	Wrinkles (2)	Rectangle (3)	$\left(\left(1\right)-\left(2\right)\right)$	$\left(\left(1\right)-\left(3\right)\right)$	$\left(\frac{\left(1\right)-\left(2\right)}{\left(2\right)}\right)$	$\left(\frac{\left(1\right)-\left(3\right)}{\left(3\right)}\right)$
1	0.005	3.19	3.113	3.112	0.077	0.078	2.47	2.51
2	0.01	3.383	3.226	3.224	0.157	0.159	4.87	4.93
3	0.015	3.58	3.341	3.337	0.239	0.243	7.15	7.28
4	0.02	3.781	3.456	3.452	0.325	0.329	9.40	9.53
5	0.025	3.986	3.571	3.566	0.415	0.42	11.62	11.78
6	0.03	4.195	3.688	3.682	0.507	0.513	13.75	13.93

## Data Availability

The original contributions presented in this study are included in the article. Further inquiries can be directed to the corresponding author.
